# A Case of Hæmorrhage after the Removal of a Canine Root

**Published:** 1849-01

**Authors:** J. A. Weaver


### A CASE OF HÆMORRHAGE AFTER THE REMOVAL OF A CANINE ROOT.

BY J. A. WEAVER, D. D. S.

*Messrs. Editors:*

IF you think the following to be of sufficient interest or service to demand the attention of the profession, you can give it through the pages of the Register.

In July last I was consulted by Mrs. D., about thirty-four years of age, in relation to her teeth and gums, and for the purpose of having some artificial teeth inserted. On examination I found the crowns of seven of the superior front teeth, including the first right bicuspis, entirely removed by decay. The roots remaining were found to be small. The gums presented rather a spungy, swollen, and livid appearance, but not excessively so.

On further examination and inquiries, I found her to be of rather an inflammatory diathesis, and highly predisposed to scrofula fugax, and scrofula americana, having had one or two attacks of this disease already within a few years past.

I ordered the extraction of the roots, and proceeded accordingly until but one remained, without any thing unusual transpiring. This last root (the left canine) manifested nothing striking in its appearance, and appeared to be somewhat loose. But in severing the gums from it, especially on the posterior side, I could sink the lancet nearly to the apex of the root; and on withdrawing it, the blood oozed out rather freer than usual; but this in a few seconds almost entirely subsided. But after removing the root, the blood flowed so freely as almost to amount to a stream; and continued bleeding at the rate of near a pint per hour (as nearly as I could judge,) for five hours. During which time, some of the most powerful styptics and astringent lotions were used (with pledgets of cotton,) such as the tincture of nutgalls, turpentine, alum, a decoction of white-oak bark, &c.; and, finally, a solution of the nitrate of silver. But all with little or no effect. The compress was omitted on account of the inconvenience it might afford the patient; but after finding all other remedies had entirely failed, I was obliged to resort to it, by first removing the coagula of blood and filling the cavity well with cotton saturated with tincture of nutgalls, and applied a large tuft of cotton over the gums. I then spread a strip of cotton cloth, from two to three inches in width, over the cotton, passed it up over the crown of the head, and tied it sufficiently tight to stop the bleeding. This bandage was worn four hours, then removed. The pledgets of cotton were left to drop out from suppuration.

I give the case without comment, and leave the reader to judge what agency the idiosyncracy of the patient had in producing hæmorrhage.

Respectfully yours,

BATAVIA, O. J. A. WEAVER.

